# A Simplified Questionnaire for the Assessment of Inorganic Arsenic Intake in a Japanese Population

**DOI:** 10.3390/ijerph17176252

**Published:** 2020-08-27

**Authors:** Jun Yoshinaga, Yuki Serizawa, Shota Suzuki, Md Hasan Al Amin, Naoko Yamada, Tomohiro Narukawa

**Affiliations:** 1 Faculty of Life Sciences, Toyo University, 1-1-1 Izumino, Itakura, Oura, Gunma 374-0193, Japan; temp13seri@gmail.com (Y.S.); baseballplayer08222@gmail.com (S.S.); hamin20072018@gmail.com (M.H.A.A.); 2 Faculty of Sport Science, Nippon Sport Science University, 7-1-1 Fukasawa, Setagaya, Tokyo 158-8508, Japan; n-yamada@nittai.ac.jp; 3 National Metrology Institute of Japan, National Institute of Advanced Industrial Sciences and Technology, 1-1-1 Umezono, Tsukuba, Ibaraki 305-8563, Japan; tomohiro-narukawa@aist.go.jp

**Keywords:** inorganic arsenic, daily intake, questionnaire, rice, hijiki

## Abstract

A simplified questionnaire was developed to assess inorganic arsenic (iAs) intake level in a Japanese population. The two page questionnaire included photographs of single serving sizes of rice and cooked hijiki (*Hizikia fusiforme*: brown algae), and asked subjects about the number of servings of rice and cooked hijiki, two predominant dietary sources of iAs in Japan, they consume in a day. Daily intake of iAs was estimated for 72 Japanese subjects using the questionnaire together with data of iAs content in rice and hijiki seaweed, and the estimated intakes were compared with actual iAs intakes of the subjects as measured for a duplicate diet using liquid chromatography–inductively coupled plasma mass spectrometry. A highly significant correlation was found between the estimated and measured intakes (*r* = 0.65, *p* < 0.001); however, the slope of regression indicated a systematic error in the intake estimation. Possible sources of error are discussed herein. It was concluded that this approach is promising if minor improvements are made to the questionnaire.

## 1. Introduction

Inorganic arsenic (iAs) is a human carcinogen. Elevated incidence of skin, lung, and bladder cancer has been found in regions of the world where the groundwater is contaminated with iAs [1]. Recently, this cancer risk has become concerning for people in noncontaminated regions who ingest iAs through their diet in the form of foods that contain naturally occurring iAs. Epidemiological studies to fully characterize the cancer risk to people in noncontaminated regions are warranted. To this end, a cost-effective exposure assessment approach is required.

In the past, total arsenic concentration in well water was used as a proxy for iAs intake in epidemiological studies of cancer in regions with contaminated groundwater; however, for the assessment of dietary iAs intake in noncontaminated regions, a different approach is required. Biomarkers of exposure, such as arsenic concentrations in urine, hair, or nails, have been suggested for this purpose [2,3]. The biomarker approach is useful in that the arsenic in these biological samples reflects internal levels; however, problems are also present. Firstly, speciation analysis is essential for the assessment of iAs intake in people who consume marine products, which contain a variety of organic arsenic species, and this requires more intensive analytical work than measuring total arsenic concentration. Secondly, urinary concentration is a suitable biomarker of recent (a few days) intake levels [4]; use of urine is not suitable for the assessment of long-term intake levels. For investigation of the epidemiology of chronic disease and cancer, assessment of long-term intake levels is required. In this regard, arsenic concentrations in hair or nails may be more suitable biomarkers of long-term intake; however, the problem of external contamination of hair [5] has not been solved, and speciation analysis of solid samples such as hair and nail, which requires solubilization of samples without decomposition/conversion of organic species, is not straightforward. These are serious unsolved problems, particularly the exposure assessment of marine-product-consuming populations, although the use of toenails has been suggested for populations in which the predominant source of As is drinking water (e.g., References [6,7]).

Food-consumption survey approaches, including food diaries, food frequency questionnaires (FFQs), etc., are promising because they require less intensive work for the assessment of intake. Generally, this approach requires a full list of the food items in which the chemical of interest can be found; however, such a list was not necessary for the assessment of iAs intake in Japan because sources of iAs are limited. Oguri et al. [8] reported in their market basket survey that rice and hijiki seaweed are the predominant sources of iAs for Japanese people. They constitute >90% of daily iAs intake of Japanese people. Therefore, assessment of rice and hijiki consumption is a simple and promising approach for the assessment of dietary iAs intake in a Japanese population.

In this paper, a simplified questionnaire was developed and its applicability was evaluated by comparison with a duplicate-diet approach.

## 2. Materials and Methods 

### 2.1. Subjects

The 72 subjects of this study were a subset of a subject group who participated in a study aiming to establish an association between iAs intake and urinary iAs and metabolite concentration [9]. The present subjects included 34 males and 38 females with a mean age of 47.3 ± 20.8 years. This study was approved by Ethical Committee of Toyo University (approval number: TU2017-005).

### 2.2. Questionnaire

**Figure 1:**
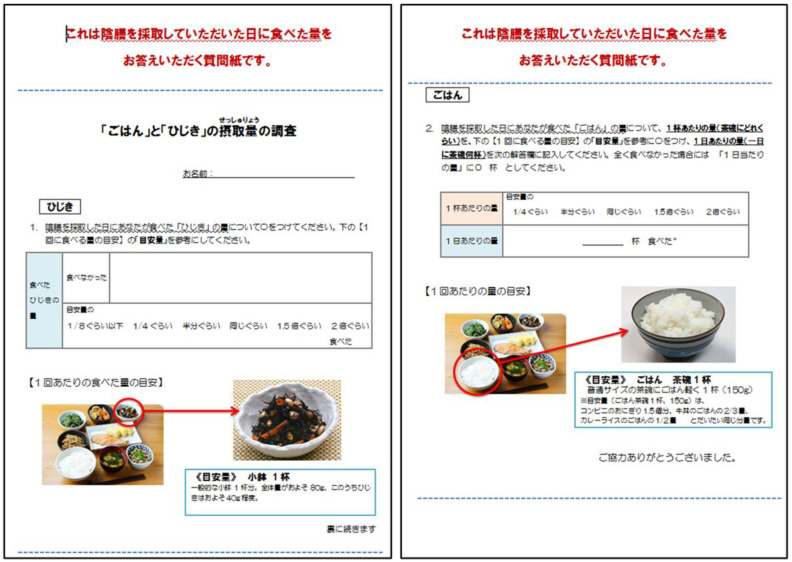
Photograph of the developed questionnaire. It is written in Japanese (because it is for application to the Japanese) on both side of a sheet with photographs of a bowl of rice and a dish of cooked hijiki to indicate standard serving size.

A simplified questionnaire was developed for this study (Figure 1). The questionnaire was printed on both sides of a sheet of paper, and the two sides asked in Japanese about the subject’s consumption of rice (brown and polished) and cooked hijiki, respectively, on the day of duplicate diet sampling. Subjects were asked about cooked hijiki consumption by the question “Did you eat cooked hijiki on the day of duplicate diet sampling?” If “Yes”, a further question asked “How much cooked hijiki did you eat?” with answer choices “<1/8, 1/4, 1/2, 1, 1.5, or 2 times the portion shown in the photograph”. The questionnaire also asked about polished and brown rice consumption with the question “How many bowls of rice did you eat on the day of duplicate diet sampling?” with approximate serving sizes quantified as “1/4, 1/2, 1, 1.5, or 2 times the portion shown in the photograph”. These were the only questions on the questionnaire. The photographs in the questionnaire indicated standard serving sizes of rice and cooked hijiki (with vegetables), i.e., 150 g and 80 g, respectively [10]. It typically took subjects less than one minute to fill out the questionnaire.

### 2.3. iAs Content of Rice and Hijiki Seaweed

Data published by the Ministry of Agriculture, Forestry and Fisheries of Japan (MAFF) on iAs content of samples of brown rice (*n* = 1200), polished rice (*n* = 6000), and hijiki seaweed (*n* = 71, water-soaked) harvested in Japan [11] were used to estimate subjects’ iAs intake based on the questionnaire. The average iAs contents of brown rice, polished rice, and soaked hijiki were 0.18, 0.12 and 3.6 mg/kg, respectively. The iAs contents of brown and polished rice are expressed on a dry weight basis, while that of soaked hijiki is on a fresh weight basis.

An additional 31 hijiki samples were purchased in Kanto District, Japan and were prepared for iAs analysis in our laboratory. The hijiki, sold in dried form, was soaked in 40 times its volume of purified water (e.g., 5 g in 200 mL) for 30 min, after which the water was discarded [12]. The soaked sample was then rinsed twice with 40 times its volume of purified water. Excess water was removed by placing the soaked hijiki on a plastic mesh for approximately 30 min. The soaked hijiki was homogenized in a food processor (Cuisinart: Tokyo, Japan), freeze-dried, and pulverized with an agate mortar and pestle.

### 2.4. Duplicate Diet Samples

Duplicate diet (DD) samples were supplied by the 72 subjects. The subjects were asked to collect all of the food and beverages they consumed over a 24 h period in polypropylene containers and bottles, and were also asked to record their menu. The DD samples were individually homogenized in a food processor, and a portion (approximately 60 g) of each homogenized sample was freeze-dried and further pulverized to make it more homogenous. The method is described in detail in our previous study [9].

### 2.5. Speciation Analysis

iAs contents of soaked and freeze-dried hijiki and freeze-dried duplicate diet samples were measured by liquid chromatography–inductively coupled plasma mass spectrometry after extraction in 0.07% hydrochloric acid + 0.01% pepsin [9]. A high performance liquid chromatography (Nanospace SI-2, Osaka Soda Co., Ltd.: Osaka, Japan) system equipped with a CAPCELL PAK C_18_ MG column (length 150 mm, internal diameter 4.6 mm, Shiseido Co., Ltd.Osaka Soda) was used for the separation of the As species, and the ICP-MS used an Agilent 7500-ce (Agilent Technologie: Tokyo, Japan) with helium as the collision gas (flow rate: 3 mL/min). The analysis was extensively validated through the analysis of certified food matrix reference materials [9] The analytical result of NMIJ CRM 7405—a Hijiki Seaweed was 10.1 ± 0.3 mg/kg (*n* = 8) (certified value: 10.5 ± 0.5 mg/kg) and that of NMIJ CRM 7502—a White Rice Flour was 0.096—0.004 mg/g (*n* = 8) (0.098 ± 0.006 mg/kg).

### 2.6. Estimation of iAs Intake Based on Questionnaire

Based on the MAFF data, the iAs content of ready-for-consumption steamed polished rice was estimated by considering the iAs lost by washing (23.2% [13]) and the average moisture content of steamed rice in Japan (60% for polished and brown [14]; 0.037 μg/g (0.12 μg/g × (1−0.232) × (1−0.60)). That of steamed brown rice was estimated to be 0.070 μg/g (0.18 μg/g × (1−0.026) × (1−0.60)). Loss of iAs from brown rice by presteam washing (2.6%) was ascertained in a preliminary experiment of this study by using 20 brown rice samples.

From the questionnaire, the number of bowls eaten by a subject was multiplied by 150 g/bowl and the iAs content of steamed rice (polished and brown), and the number of dishes of cooked hijiki was multiplied by 40 g/dish and the mean iAs content of soaked hijiki. The 40 g hijiki seaweed/80 g cooked hijiki was based on a previous study in which percentage of hijiki seaweed in cooked hijiki was measured for 14 cooked hijiki samples prepared in Japanese households [15]. The sum of iAs from rice and cooked hijiki was regarded as the estimated iAs intake.

## 3. Results

Of the 72 subjects of this study, two did not return the questionnaire and a further five subjects failed to fully complete it, leaving 65 subjects for analysis. Of the 65 subjects, 59 ate polished rice on the day, 6 ate brown rice, and 18 ate cooked hijiki. Table 1 shows the number of bowls of rice (polished and brown) and the number of dishes of cooked hijiki the subjects reported eating on the day of duplicate diet sampling.

The mean ± standard deviation of the iAs concentration in 31 soaked hijiki samples was 1.81 ± 1.19 μg/g. The mean iAs concentration reported in the MAFF data was 3.6 μg/g (*n* = 71); therefore, weighted average iAs concentration in soaked hijiki was calculated to be 3.1 μg/g (*n* = 102).

The mean estimated iAs intake of the 65 subjects was 39.6 ± 51.6 μg/day. Median (min–max) was 16.7 (0–237) μg/day. Meanwhile, the measured daily intake from the duplicate diets of the 65 subjects was 38.4 ± 36.2 μg/day. Median and min–max were 28.9 and 0.6–186 μg/day, respectively. The correlation between the estimated and measured iAs intake was significant (*r* = 0.645, *p* < 0.001) and regression equation was (measured value) = 0.452 × (estimated value) + 20.4. The 95% confidence interval (CI) values of the slope and intercept were 0.317–0.587 and 11.7–29.1, respectively.

**Figure 2:**
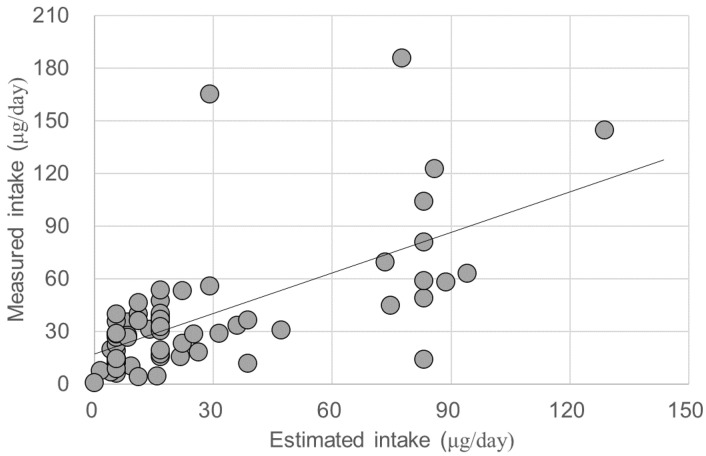
Scatter plot of estimated and measured inorganic arsenic intakes. Estimated intake (horizontal axis) was calculated using the measured iAs content of hijiki seaweed (1.81 mg/kg, *n* = 31) and the iAs content of rice according to MAFF, Ministry of Agriculture, Forestry and Fisheries of Japan, data and measured intake (vertical axis) was measured against iAs intake based on the duplicate diet. Correlation coefficient was *r* = 0.647 (*p* < 0.001) and regression equation was (measured value) = 0.768 × (estimated value) + 17.1.

Since a significant difference was observed between the MAFF mean (3.6 μg/g) and our measured value (mean: 1.81 μg/g), the correlation analysis and regression analysis were carried out using our measured hijiki iAs only. When the iAs content of hijiki was replaced with 1.8 μg/g, the mean estimated iAs intake decreased to 27.6 ± 30.5 μg/day (median: 16.7 μg/day). The correlation coefficient was *r* = 0.647 and regression equation was (measured value) = 0.768 × (estimated value) + 17.1 (Figure 2). The 95% CI values of the slope and intercept were 0.540–0.996 and 7.8–26.5, respectively.

## 4. Discussion

The highly significant correlation between estimated and measured iAs intake (*r* = 0.645 or 0.647) indicated the usability of the developed questionnaire for the assessment of iAs intake. It took only <1 min to fill the questionnaire; therefore, it allowed assessment of intake without substantial burden to the subjects and could be applied to a large number of subjects without much burden for researchers.

However, the slope of regression between the estimated and measured iAs intake was significantly lower than unity and the intercept was significantly higher than 0 (20.4 or 17.1) indicating overestimation of the intake by the questionnaire. One of the probable reasons for this overestimation is related to the iAs content of the hijiki seaweed we used for the iAs intake estimation. Since the iAs content of hijiki is high and the iAs intake of the subjects who ate cooked hijiki was significantly elevated, i.e., one dish of cooked hijiki contained >120 μg of iAs while one bowl of steamed rice contained 6 μg, it was assumed that the iAs concentration in hijiki would greatly affect the slope. We first used the reported and measured weighted mean concentrations (3.1 μg/g) of iAs in hijiki. The mean of the measured concentrations (1.8 μg/g) was half of the MAFF mean (3.6 μg/g). When we used only our measured iAs concentration for hijiki, the slope increased to 0.768 (95% CI: 0.540–0.996). The reason(s) for the difference between the MAFF mean and ours was not clear. Since the soaking method used for hijiki samples in the MAFF investigation was not published, it is possible that differences in the soaking process could be the reason. Soaking significantly reduces the iAs content of hijiki [12]. It may be more appropriate to use our measured hijiki iAs concentration for estimation because the soaking method employed in the present study [12] was close to that used in general households in Japan.

Even when we replaced the iAs content of hijiki seaweed with our measured mean, the slope (0.768) was still lower than unity and intercept was still high (17.1, 95% CI: 7.8–26.5) (Figure 2). This indicated the presence of other reason(s) for overestimation in the estimated intake. The moderate number of subjects included in this study and variability in the iAs content of the rice and hijiki actually consumed by the subjects may have contributed to this bias. Another possible reason is that subjects’ misjudgment of the cooked hijiki serving size shown in the photograph in the questionnaire. The standard size of a rice bowl is commonly understood by Japanese subjects because rice is a staple food. In contrast to the clear and common image of the size of a rice bowl, the size of a dish of cooked hijiki could be more variable. This could result in misjudgment of the serving size. The inclusion of a ruler in the photograph of a dish of cooked hijiki might help subjects to properly recognize the indicated serving size.

Cooked hijiki is usually prepared by frying and boiling hijiki seaweed and soy beans or vegetables, and the abundance of hijiki seaweed in cooked hijiki can vary from one household to another based on their recipe: our unpublished data on the proportion of hijiki seaweed in 14 cooked hijiki samples prepared in Japanese households varied from 18% to 73%. In the present study, we assumed that proportion of hijiki seaweed in cooked hijiki was 50.4% by taking the mean of the 14 samples [15] for the estimation. Therefore, the variable abundance of hijiki seaweed in cooked hijiki could also contribute to the inconsistency.

Thus, for more accurate quantitative estimation of the iAs intake of Japanese subjects, the establishment of a representative iAs content of hijiki seaweed and refinement of hijiki consumption estimation in the questionnaire are indicated.

Assessment of the daily iAs intake of individuals in a subject population is a prerequisite of epidemiological research related to the occurrence of chronic diseases, including cancers. Measurement of iAs in diet samples collected from subjects—a duplicate diet—is a reliable approach for quantification of iAs intake, but the cost- and labor-intensiveness makes this approach unsuitable for epidemiological studies. The urinary biomarker approach may be less labor-intensive; however, analytical sensitivity to iAs at the levels encountered in urine samples from the general population is sometimes insufficient, and individual metabolic differences may pose a problem. In addition, the duplicate-diet approach and urinary biomarker approach are not suitable for the assessment of long-term intake levels of iAs because they are basically methods of assessment for one day or recent intake levels. In this regard, the present questionnaire approach also provided one day iAs intake level, as the duplicate-diet approach did; however, since this questionnaire is simple and easy to fill, it is possible to repeat the assessment multiple times with the same subjects over a long-term period to gather information on long-term (months or years) intake levels. Alternatively, the present questionnaire could be modified to ask about the number of standard serving sizes of rice and cooked hijiki the subject usually consumes and their frequency for the assessment of habitual intake level, like a FFQ. Use of biomarkers such as hair and nails may also be applicable for long-term intake assessment, although only if the external contamination problem is solved and speciation analytical methods for these sample matrixes are established to specifically evaluate iAs intake levels.

Although the questionnaire (Figure 1) was developed exclusively for application to a Japanese population, the approach presented in this study could be applied to other populations without iAs contamination of groundwater. Some conditions are required for the application of this approach: (1) dietary source(s) of iAs in that population is clearly identifiable, presumably based on a reliable market basket survey; (2) the number of sources is preferably small, so that questionnaire can be concise; (3) representative iAs contents of the source foods are available. Considering the increased worldwide concern over the potential health effects of dietary iAs exposure, more and more attention will be directed to the assessment of dietary iAs exposure in many countries and that effort will enable application of the simplified questionnaire approach suggested in this study to iAs exposure assessment in other populations.

## 5. Conclusions

We developed a simple questionnaire for estimating iAs intake of subjects by asking about consumption of rice and hijiki for possible application in epidemiological research. The highly significant correlation between estimated and measured iAs intake indicated this approach to be promising, although some bias was also found. It was concluded that even such a simple questionnaire can have the potential to quantitatively estimate the iAs intake of subjects, although some more refinement of the questionnaire seems to be necessary. This questionnaire was developed for application in a Japanese population, but this approach could also be applicable to other populations if the source(s) of dietary iAs is clearly defined in the population of interest.

## Figures and Tables

**Table 1:** Number of bowls of rice and of dishes of cooked hijiki the subjects ate in a day.

	Number of Bowls		Number of Dishes
	Polished Rice	Brown Rice	Cooked Hijiki
Mean (SD)	1.7 (1.2)	0.11 (0.47)	0.23 (0.41)
Median (min–max)	1.5 (0–4.5)	0 (0–3)	0 (0–1.5)
